# The genomic basis of the *Streptococcus thermophilus* health-promoting properties

**DOI:** 10.1186/s12864-022-08459-y

**Published:** 2022-03-16

**Authors:** Emeline Roux, Aurélie Nicolas, Florence Valence, Grégoire Siekaniec, Victoria Chuat, Jacques Nicolas, Yves Le Loir, Eric Guédon

**Affiliations:** 1 INRAE, Institut Agro, STLO, Rennes, France; 2 Université de Lorraine, CALBINOTOX, Nancy, France; 3 Université de Rennes, INRIA, Campus de Beaulieu, Rennes, France

**Keywords:** Lactose, Galactose, GABA, Comparative genomics, Pangenome, PrtS, Vitamin, Conjugated linoleic acid, Bacteriocin

## Abstract

**Background:**

*Streptococcus thermophilus* is a Gram-positive bacterium widely used as starter in the dairy industry as well as in many traditional fermented products. In addition to its technological importance, it has also gained interest in recent years as beneficial bacterium due to human health-promoting functionalities. The objective of this study was to inventory the main health-promoting properties of *S. thermophilus* and to study their intra-species diversity at the genomic and genetic level within a collection of representative strains.

**Results:**

In this study various health-related functions were analyzed at the genome level from 79 genome sequences of strains isolated over a long time period from diverse products and different geographic locations. While some functions are widely conserved among isolates (e.g., degradation of lactose, folate production) suggesting their central physiological and ecological role for the species, others including the tagatose-6-phosphate pathway involved in the catabolism of galactose, and the production of bioactive peptides and gamma-aminobutyric acid are strain-specific. Most of these strain-specific health-promoting properties seems to have been acquired via horizontal gene transfer events. The genetic basis for the phenotypic diversity between strains for some health related traits have also been investigated. For instance, substitutions in the *galK* promoter region correlate with the ability of some strains to catabolize galactose via the Leloir pathway. Finally, the low occurrence in *S. thermophilus* genomes of genes coding for biogenic amine production and antibiotic resistance is also a contributing factor to its safety status.

**Conclusions:**

The natural intra-species diversity of *S. thermophilus*, therefore, represents an interesting source for innovation in the field of fermented products enriched for healthy components that can be exploited to improve human health. A better knowledge of the health-promoting properties and their genomic and genetic diversity within the species may facilitate the selection and application of strains for specific biotechnological and human health-promoting purpose. Moreover, by pointing out that a substantial part of its functional potential still defies us, our work opens the way to uncover additional health-related functions through the intra-species diversity exploration of *S. thermophilus* by comparative genomics approaches.

**Supplementary Information:**

The online version contains supplementary material available at 10.1186/s12864-022-08459-y.

## Background

*Streptococcus thermophilus* (STH) is a nonpathogenic Gram-positive bacterium belonging to the phylum *Firmicutes* and family *Streptococcaceae*. It belongs to the salivarius group of the Viridians streptococci, which includes two other species, *Streptococcus salivarius* and *Streptococcus vestibularis* [1]. These two species are human commensal bacteria, whereas the environmental reservoir of STH has not been yet identified [2]. STH that has a long history of use in dairy fermentations grows spontaneously in numerous traditional dairy products and is believed to persist in the farm environment [3–5]. STH also belongs to the heterogeneous group of lactic acid bacteria (LAB), that includes notably species from the genera *Lactococcus*, *Lactobacillus*, *Enterococcus*, *Carnobacterium*, *Leuconostoc*, *Oenococcus*, and *Pediococcus* and plays a crucial role in food fermentation. STH is widely used as a starter in the dairy industry as well as in many traditional fermented products including yogurt and various soft (e.g., Mozzarella), hard (e.g., Cheddar, Emmental, Parmigiano, Pecorino), surface ripened (e.g., Gruyère, Appenzeller, Tête de Moine) and mould ripened cheeses (e.g., Gorgonzola) [6, 7]. STH is also found in dairy products spontaneously fermented such as diverse fermented milks in Mongolia [8] and Ragusano cheese in Sicily [9]. In addition, STH can participate to fermentation of leguminous such as soy juice [10, 11] and milk kefir grains [12, 13].

STH is a clonal species that emerged only recently on the evolutionary timescale (3000–30,000 years ago), from a commensal ancestor of the salivarius group [14]. Its remarkable adaptation to grow in milk, a narrow and well-defined niche, has resulted to its genome shaping through loss-of-function events and horizontal gene transfers (HGT) [15–17]. Approximately 10% of the STH predicted proteome are pseudogenes [15]. Most of them correspond to original functions being unnecessary for growth in milk such as proteins involved in carbohydrate metabolism [16, 17] and surface-exposed proteins that are involved in host-bacteria interactions [2, 15, 18]. HGT events have contributed substantially to the genomic plasticity, population evolution and adaptation of the species to the milk environment. The genomic regions acquired include those encoding industrially important phenotypic traits, such as the production of bacteriocins and exopolysaccharides, restriction-modification systems, oxygen tolerance, amino acid metabolism and milk-protein degradation [16, 17, 19–22].

Due to the technological properties and commercial importance of STH, its physiological and metabolic properties have been the subject of numerous studies such as rapid acidifying ability, fast growth, proteolytic ability, production of exopolysaccharides (EPS) and phage resistance [16, 17, 19, 23, 24]. In addition to its technological importance, STH has also gained interest in recent years as a probiotic bacterium [25]. Many commercial probiotic products available on the market all over the world contain STH [26], although its ability to survive the stomach environment can be questionable for a probiotic [25, 27]. The presence of STH in fermented products has been linked to various beneficial effects on human health, including alleviation of lactose intolerance [28], inhibition of pathogens [29], prevention of diarrhea [30, 31], modulation of inflammation and cytokine production [32–36], reduction of uremia [37], decrease of total cholesterol and low-density lipoprotein cholesterol [38, 39], prevention of the development of insulin resistance and improvement of glycemic parameters [40, 41], reduced risk of developing certain cancers [42–44], prevention or treatment of inflammatory gut diseases [14, 45–47] and maintain gut homeostasis [48]. Although most of the bacterial factors and mechanisms behind these beneficial effects remain unknown, some, however, are being elucidated. For instance, the LacZ beta-galactosidase released by STH and involved in lactose catabolism may play a role in colorectal cancer prevention [44]. Indeed, secreted LacZ inhibits cell proliferation, lowers colony formation, induces cell cycle arrest, promotes apoptosis of colorectal cancer cells in vitro and reduces tumor formation in mice. The anti-cancer effects of STH is partially mediated through the ability of LacZ to liberate galactose that interferes with energy homeostasis to activate oxidative phosphorylation and downregulate the Hippo pathway kinases [44]. The EPS from strain CRL1190 stimulate epithelial cell regeneration and immunological innate defense mechanisms in vitro [49] and prevent chronic gastritis induced by acetylsalicylic acid in mice [50, 51]. The EPS from strain AR333 exhibit immuno-stimulant effect in vitro [52]. The EPS-mediated beneficial effects are, however, strain dependent [50]. Lactate produced by STH in the digestive tract of rodents promotes mucus pathway and modulates colon epithelium [53, 54]. Lactate also inhibits growth of pathogens, participates in the trophic chain between microbial communities and exhibits anti-proliferative effects [55–57]. Anti-oxidative components produced by STH including reduced glutathione could be involved in the beneficial effects of STH fermented milks on cardiovascular disease risk factors and hypercholesterolemia, notably via their activity against low-density lipoprotein oxidation [58, 59]. Proteolysis of caseins by yogurt bacteria including STH liberates bioactive peptides that, among others, stimulate mucin gene expression and secretion in vitro and enhance the number of goblet and Paneth cells along the small intestine and the expression of intestinal mucins and antibacterial factors in vivo [60]. Also, it has been proposed that the positive effect of STH strain ST4 on mice with 5-fluorouracil-induced mucositis is attributed to an increase in the content of fecal short chain fatty acid (SCFA) acetate, in correlation with maintenance of inflammatory homeostasis and preservation of intestinal permeability [61]. Finally, folate produced by STH would be involved in the reduction of inflammation in rodent models of induced mucositis [46, 62–64]. The development of dairy fermented products naturally enriched in health-promoting metabolites produced by STH during fermentation such as gamma-aminobutyric acid (GABA, the major inhibitory neurotransmitter in the central nervous system) and folate (the essential vitamin B9) has also been investigated [65–68]. Despite these numerous potential beneficial effects, however, the only STH-related health claim recognized by European Food Safety Authority (EFSA) is linked to the live yogurt culture consumption on lactose maldigestion [69].

**Fig. 1:**
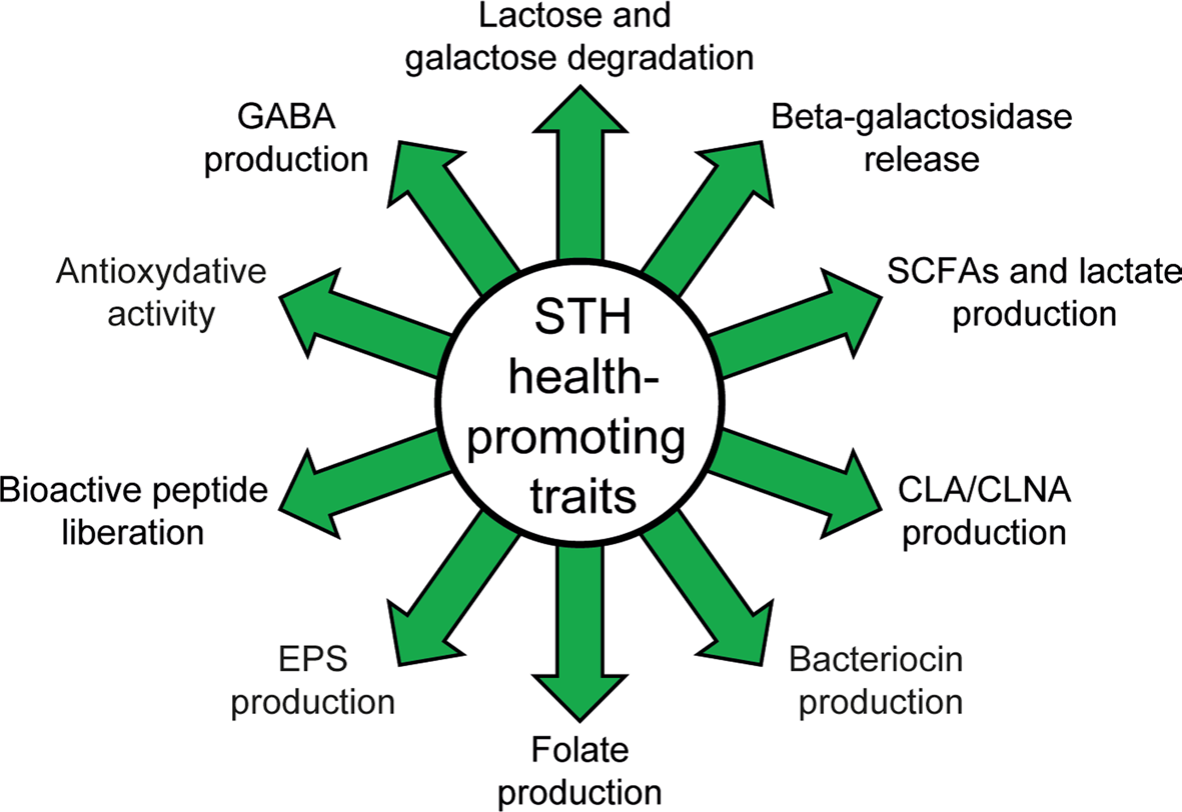
Health-promoting properties of *S. thermophilus*. SCFAs, short chain fatty acids; CLA, conjugated linoleic acid; CLNA, conjugated linolenic acid; EPS, exopolysaccharides; GABA, gamma-aminobutyric acid

Most of these functionalities are strain-dependent suggesting that the wide range of health-promoting properties of STH results in a high degree of variation in the content of strain genomes. Analysis at the genome level is, thus, required to better understand strain features. Thanks to the democratization of low-cost sequencing systems, the number of sequenced bacterial genomes has boomed in the last decade. For instance, among the genus *Streptococcus*, around 31,000 *Streptococcus pneumoniae*, 5000 *Streptococcus pyogenes*, 3000 *Streptococcus agalactiae*, 350 *S. salivarius*, and 16 *Streptococcus macedonicus* genomic sequences are available in the NCBI genome database (https://www.ncbi.nlm.nih.gov/genome/). The first complete genome sequence of STH dates back to 2004 [15] and ever since more than 100 genomes are now publicly available at NCBI. Among them, 50% were deposited in the last 2 years showing the growing interest for STH. The purpose of this study is to explore the health-promoting properties of STH and their diversity at the genomic and molecular level across strains. To this aim, a comparative genomic study of 79 representative strains was performed on bacterial activities known to be beneficial on host physiology. They can result from the ability of STH to produce compounds beneficial for host (e.g., lactate, SCFAs, GABA, EPS, folate, bacteriocins, glutathione), to liberate enzymes that can strengthen the host capability (e.g., LacZ, antioxidative enzymes), to metabolize food components that can be harmful to sensitive people (e.g., lactose, galactose) and to release beneficial breakdown products from food components (e.g., bioactive peptides) (Fig. 1). We also evaluated the presence of genes coding for undesirable traits such as production of biogenic amines and antibiotic resistance in STH genome sequences. Finally, although STH can be considered to be a probiotic, probiotic traits were not included since genetic determinants are still elusive and the probiotic character of STH is often determined in association with other bacteria [70–72].

## Results & discussion

### General genomic features of STH

**Fig. 2:**
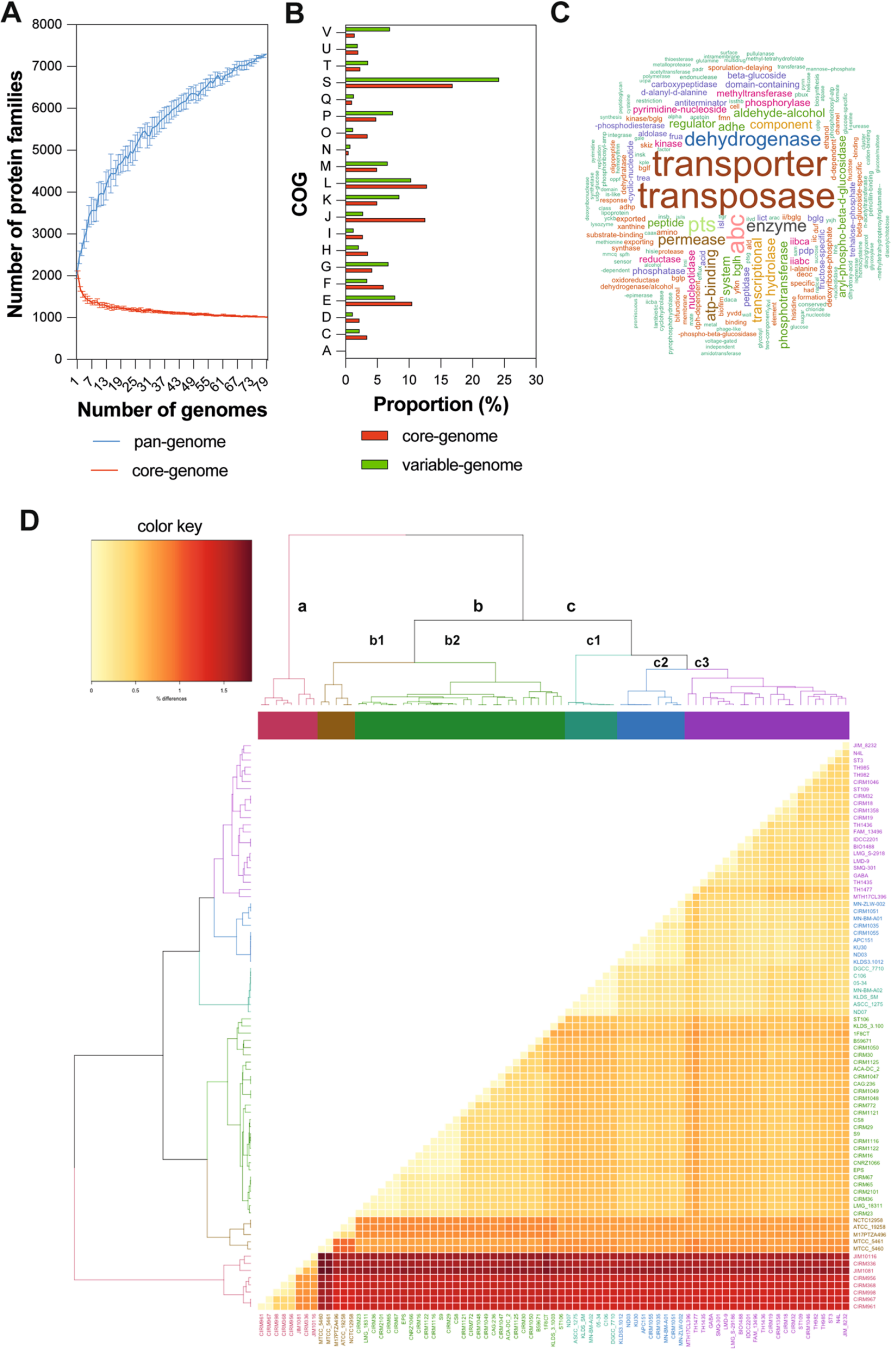
Genomic and phylogenomic features of *S. thermophilus*. **A** Core and pan-genome size evolution in *S. thermophilus* isolates according to the number of genomes. Each point represents the number of protein families conserved between genomes. All of the points are plotted as a function of the strain number (x). The deduced pan-genome size is y = 1920.3 × ^0.3052^ (R2 = 0.9972). **B**. The distribution of core and accessory proteins according to the COG classification. The *x*-axis indicates the percentage of proteins in various COG categories. (A) RNA processing and modification; (C) Energy production and conversion; (D) Cell cycle control, cell division, chromosome partitioning; (E) Amino acid transport and metabolism; (F) Nucleotide transport and metabolism; (G) Carbohydrate transport and metabolism; (H) Coenzyme transport and metabolism; (I) Lipid transport and metabolism; (J) Translation, ribosomal structure and biogenesis; (K) Transcription; (L) Replication, recombination, and repair; (M) Cell wall/membrane/envelope biogenesis; (N) Cell motility; (O) Posttranslational modification, protein turnover, chaperones; (P) Inorganic ion transport and metabolism; (Q) Secondary metabolites biosynthesis, transport, and catabolism; (S) Function unknown; (T) Signal transduction mechanisms; (U) Intracellular trafficking and secretion; (V) Defense mechanisms. **C** Word cloud of pseudogenes annotations. Annotation of pseudogenes provided by MicroScope was filtered to remove non-informative words (see Methods section for the complete list of removed words). Word size is calculated accordingly to its occurrence. **D** Heatmap showing the pairwise percentage of differences (% differences) between core proteome of the 79 STH strains. Strains were clustered into three clusters (a, b, c). Subgroups within the clusters are also identified (b1, b2, c1, c2, and c3)

Among the STH available genomes in NCBI database in January 2020, 45 were selected according to their finishing level to explore the STH health-promoting properties: 27 genomes have been fully sequenced, 8 have been completely sequenced at the chromosome level, and the remaining 10 genomes have finished draft (Table S1). We completed this dataset with the complete genome sequences of strains from CIRM-BIA collection (31 genomes) and MicroScope database (3 genomes). The 79 genome sequences selected correspond to strains isolated over a long time period (1954 to 2018) from diverse products (including cheeses, yogurts, fermented milks, starters, kefir grains, and digestive tract of marine fish), and different geographic locations (19 countries) providing a good sample of the overall species diversity of STH (Table S1). The complete genome sequences (*n* = 68) range in size from 1.73 (ACA-DC 2) to 2.10 Mb (NCTC12958) with a mean GC content of 39%. Small plasmids ranging in size from 3.17 to 4.45 kb are detected in only 10 strains: 8 strains contain one plasmid, and the others two plasmids. The ANI (Average Nucleotide identity) values that ranged from 96.95 to 100% between a strain pair with a mean of 98.63 ± 0.63 confirmed that the 79 strains selected belong to the same species (Fig. S1). The number of predicted CDS (coding DNA sequence) in each complete genome ranges from 2033 (ACA-DC 2) to 2513 (M17PTZA496) with an average of 2170 + 101 CDS per genome (median = 2150). Note that the number of predicted CDS in the incomplete genomes is slightly lower with an average of 2092 + 160 CDS (median = 2109). The pan-genome of STH estimated on 79 strains contains 7298 protein families (170,341 proteins), while 1011 protein families (86,189 proteins) constitute the core genome. It is interesting to note that the STH pan-genome is larger than that of *Listeria monocytogenes*, *Streptococcus pneumoniae* and *Staphylococcus aureus*, among others, although its genome size is smaller, pointing out the higher genetic diversity and adaptability of the species likely related to its domestication and genome evolution by HGT [73]. The accessory genome, i.e. the set of proteins absent in some genomes but present in two or more genomes, is composed of 6287 protein families (84,202 proteins). The number of strain-specific proteins (i.e. the set of proteins present in a single genome) ranges from 0 to 199 with an average of 28 unique proteins per genome. Analysis of the core and pan-genome size evolution as a function of the extent of available genomes showed opposite patterns (Fig. 2A). While the core genome curve (in red) tended to saturate, indicating that the set of protein families shared by all strains was mostly captured with the 79 selected genomes, the pan-genome curve (in blue) continuously increased with the addition of new genomes. This result shows that the STH pan-genome can be considered as open and likely reflects the life of the species in a narrow niche but with multiple ways of exchanging genetic material to guarantee its adaptation to fluctuating environments [74]. This result is also supported by the fact that almost all isolates still have specific proteins (Table S1 and S2). The fact that new protein families will always continue to be identified with additional genomes implies that the genomic diversity of the species has only been partially discovered. Distribution of core and accessory proteins in COG (Clusters of Orthologous Groups) categories is not homogeneous. Indeed, the proteins involved in RNA processing and modification (A), translation, ribosomal structure and biogenesis (J), posttranslational modification, protein turnover, chaperones (O), energy production and conversion (C), lipid transport and metabolism (I), cell cycle control, cell division, chromosome partitioning (D), replication and repair (L), amino acid metabolism and transport (E), nucleotide transport and metabolism (F) and coenzyme transport and metabolism (H) were better represented in the core genome than the accessory genome (Fig. 2B). Inversely, proteins involved in defense (V), transcription (K), secondary metabolites biosynthesis, transport, and catabolism (Q), cell wall / membrane / envelop biogenesis (M), signal transduction (T), cell motility (N), carbohydrate transport and metabolism (G), and inorganic ion transport and metabolism (P) were better represented in the accessory genome than in the core genome (Table S2). These accessory functions, which notably include bacteriocin synthesis, transcriptional factors, cell surface proteins and sugar metabolism, featured the life history traits of STH and its adaptation to dairy environment [2, 16, 17]. Finally, one genomic feature of STH is the marked level of gene decay [15]. Depending on the gene prediction used, the estimated number of pseudogenes represented on average from 5 to 10% of the STH predicted proteome (Table S1). These pseudogenes correspond to genes coding mostly for transporters (e.g., ABC transporter, PTS system, permease …), enzymes (e.g., dehydrogenase, metalloprotease, oxidoreductase, phosphatase, N-acetyltransferase, endonuclease, …), mobile element proteins (e.g., transposase) and regulatory systems (transcriptional, regulator, helix-turn-helix, histidine kinase, …) (Fig. 2C).

Recently, the phylogenomic relationship between 23 STH strains based on their core genome showed a clustering of strains into two main groups [19]. A complete linkage clustering was performed from the core genome of the 79 strains. Eighty-nine percent of the strains were present in these two groups (Cluster b and c, Fig. 2D). The inclusion of more strains, however, allowed to refine the contours of the clusters by subdividing them into two to three subgroups of strains. In addition, our analysis also revealed an additional cluster composed of eight strains, very distant from the others. The presence/absence heat map of the accessory proteins of strains also supported the existence of this additional cluster (Fig. S2). Except this additional cluster which is composed of strains mostly isolated in Italia, overall no clear association between the type of products, the year of isolation or the geographic origin of the strain and their phylogeny were evidenced. Overall, these genomic analyses pointed out that the STH population is likely more diverse than that expected and a substantial part of the STH functional potential still defies us and remains to be explored.

### Lactose and galactose metabolism

**Fig. 3:**
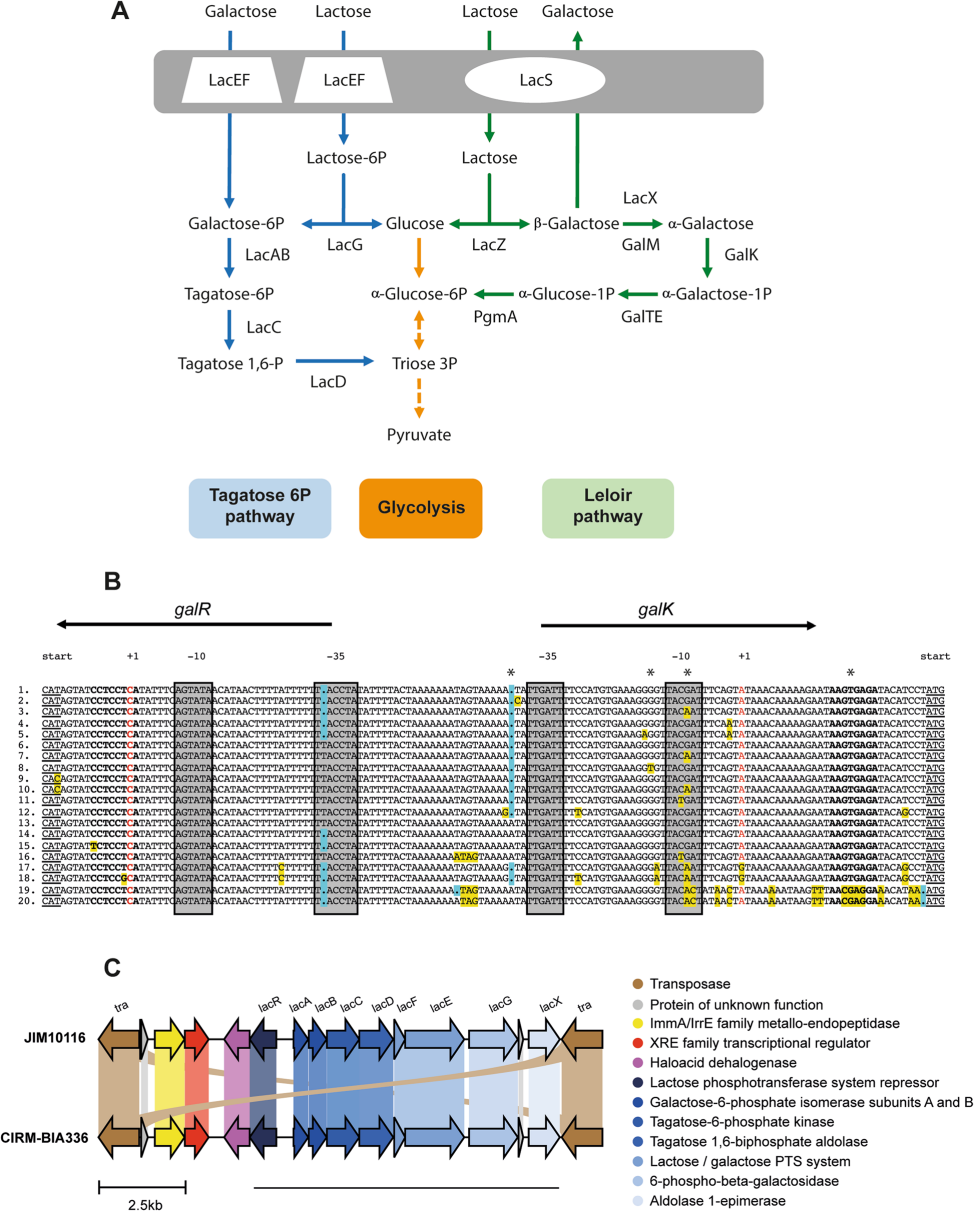
Metabolism of galactose and lactose in *S. thermophilus*. **A** Schematic representation of the main steps of lactose and galactose catabolism in STH. LacEF, lactose/galactose PTS system; LacG, 6-phospho-beta-galactosidase; LacD, tagatose 1,6-biphosphate aldolase; LacC, tagatose-6-phosphate kinase; LacAB, galactose-6-phosphate isomerase subunits A and B; LacS, lactose permease; LacZ, beta-galactosidase; GalM, galactose mutarotase; LacX, aldolase 1-epimerase; GalK, galactokinase; GalT, galactose-1-phosphate uridylyltransferase; GalE, UDP-glucose 4-epimerase; PgmA, phosphoglucomutase. **B** Nucleotide sequences alignment of the *galR*-*galK* intergenic region of various STH strains. Promoter sequences were annotated according to Vaughan et al. (2001) and Vaillancourt et al. (2002). The start codon of both *galR* and *galK* is underlined. The − 35 and − 10 sequences are grey-boxed and the ribosome binding sites are in bold. The transcription starting sites are in red. Nucleotide substitutions and indels are yellow- and blue-boxed, respectively. Stars indicate point mutations in the *galK* promoter region of Gal+ mutants. 1: Promoter sequence of strains DGCC 7710, 05–34, C106, ND07, MN-BM-A02, ASCC 1275, N4L, MTH17CL396, JIM8232, CIRM-BIA1048, CIRM-BIA1049, ST109, CAG:236, CIRM-BIA1047, MTCC 5461 and CIRM-BIA772; 2: Promoter sequence of strain CIRM-BIA1121; 3: Promoter sequence of strain B59671; 4: Promoter sequence of strain TH982; 5: Promoter sequence of strain 1F8CT; 6: Promoter sequence of strains LMG 18311, BIO1488, KU30, KLDS3.1012, CIRM-BIA1125, CIRM-BIA1116, GABA, LMG S-29186, LMD-9, CIRM-BIA1051, CIRM-BIA1055, CIRM-BIA2101, CIRM-BIA67, CIRM-BIA1050, CIRM-BIA36, TH985, FAM 13496, CIRM-BIA16, IDCC2201, ACA-DC 2, CIRM-BIA18, CIRM-BIA32, CIRM-BIA1358, CIRM-BIA19, APC151, CIRM-BIA1122, MN-ZLW-002, ND03, SMQ-301, MN-BM-A01, and S9; 7: Promoter sequence of strain EPS; 8: Promoter sequence of strain CIRM-BIA65; 9: Promoter sequence of STH CNRZ1066; 10: Promoter sequence of *S. thermophilus* CS8; 11: Promoter sequence of strain CIRM-BIA1046; 12: Promoter sequence of strain TH1477; 13: Promoter sequence of strains CIRM-BIA23, CIRM-BIA29, CIRM-BIA30, and CIRM-BIA1035; 14: Promoter sequence of *S. thermophilus* KLDS SM; 15: Promoter sequence of *S. thermophilus* KLDS 3.1003; 16: Promoter sequence of strain ST3; 17: Promoter sequence of strain TH1436; 18: Promoter sequence of strains M17PTZA496, ATCC 19258, and NCTC12958; 19: Promoter sequence of strains JIM1081, CIRM-BIA961, CIRM-BIA967, JIM10116, and CIRM-BIA336; 20: Promoter sequence of strains CIRM-BIA368, CIRM-BIA956, and CIRM-BIA998. **C** Genetic organization of genes coding for the tagatose 6P pathway in strain JIM10116 and CIRM-BIA336. The lengths of genes (pentagons) and intergenic regions are drawn to scale. Blue-colored pentagons code for proteins involved in galactose/lactose assimilation. The underlined part of the cluster corresponds to the region with high nucleotide identity (> 96%) with gene clusters found in various *Enterococcus* and *Lactococcus* species

As mentioned above, the ability of STH to ferment carbohydrates, notably lactose, contributes to its beneficial effects via the production of lactate, the major end-product of carbohydrate fermentation able to modulate mucus pathway and colon epithelium [53, 54], and the synthesis of LacZ (beta-galactosidase, lactase), a catabolic enzyme with potential role in colorectal cancer prevention [44]. The lactose and galactose metabolism of STH can also help to manage metabolic disorders associated to lactose and galactose intolerance. Indeed, the primary function of STH in dairy fermentation is the rapid acidification of milk that leads to a modification of the physico-chemical properties of milk and an inhibition of the growth of spoilage and pathogenic microorganisms. Milk acidification results from the production of lactic acid by fermentation of lactose, the major carbohydrate in milk. Lactose fermentation by STH and other LAB that contributes, therefore, to manufacture low-lactose and bacterial lactase-containing dairy products and the ability of STH to reduce amount of lactose in the small intestine by producing an active LacZ are suitable for lactose intolerant people [75]. Note that over 60% of the human population has a reduced ability to digest lactose [76]. STH imports lactose through the LacS permease, which functions as a lactose/galactose antiporter (Fig. 3A). Lactose is further cleaved by LacZ (EC 3.2.1.23) into glucose and galactose that are metabolized by the glycolytic pathway and Leloir pathway, respectively. The genome of most STH strains contained intact copy of *lacS* and *lacZ* (Table S3). Catabolism of lactose results in production of lactate and other end products, while galactose serves to synthesize polysaccharides, teichoic acids and nucleotide sugars. Galactose can also enter glycolysis through phosphoglucomutase (PgmA; EC 5.4.2.2) and Leloir pathway. Although all the strains contain the genes coding for the Leloir pathway (GalKTEM) and PgmA (Table S3), most STH strains are unable to ferment galactose, which is generally expelled into the medium [77–80]. Presence of galactose in dairy products is harmful for individuals with galactosemia, a genetic metabolic disorder that affects their ability to metabolize galactose properly. Therefore, development of dairy products with low galactose level has been investigated [81].

The low expression level of *galKTEM* operon and the low activity of the galactokinase GalK represent the major limiting steps for galactose utilization in STH. Spontaneous STH mutants able to ferment galactose (Gal+ mutants) have been isolated [82]. They contain mutations at three positions in the *galK* promoter region including substitutions and a single-base-pair insertion that lead to increased *galK* expression. Among them, one substitution located in the − 10 box of *galK* promoter region was also independently found in a spontaneous Gal+ mutant [83] and associated with the Gal+ phenotype of various STH wild-type strains indicating that the *galK* promoter region plays a crucial role in galactose phenotype of the strains [77–85]. The activity of GalK is also a limiting step for galactose metabolism and substitutions in the ribosome binding site of *galK* increase GalK activity [79, 82, 83]. Analysis of the *galR*-*galK* intergenic region from 79 STH strains showed numerous nucleotide substitutions and single-base-pair insertions/deletions (Fig. 3B). Notably, 15 strains have the same nucleotide A insertion preceding the – 35 region of Gal+ mutants, one strain has the same G-to-T substitution preceding the - 10 box of Gal+ mutants, 15 strains have the same nucleotide G-to-A substitution in the − 10 box of Gal+ mutants*,* and 8 strains have several nucleotide substitutions in the *galK* ribosome binding site [79, 80, 82, 83]. A possible link between these different nucleotide variations and the ability of these strains to catabolize galactose remains to be established.

Finally, the reduction of accumulation of galactose in fermented milks can also be achieved through the use of strains able to ferment galactose through the tagatose-6-phosphate pathway (T6P) [86]. Such strains uptake lactose or galactose via PTS systems resulting in the formation of lactose/galactose-6-phosphate, which is further catabolized by T6P pathway [87]. Interestingly, two STH strains, CIRM-BIA336 and JIM10116, contained genes coding for the LacEF PTS system and the LacABCDG enzymes of the T6P pathway (Fig. 3A). These genes were clustered into a single genomic region flanked by a transposase and of approximately 10 kb which is 99% identical between the two strains (Fig. 3C). The region from *lacR* to *lacX* also shared high nucleotide identity (> 96%) with plasmid or genomic regions from numerous strains of various *Enterococcus* and *Lactococcus* species. The cluster also encoded LacX aldolase 1-epimerase (EC 5.1.3.3), which catalyzes the conversion of β-glucose and β-galactose into their α-isomers, which might be important for the efficient catabolism of galactose notably through the Leloir pathway. The low occurrence of this cluster in the STH population (2/79)*,* the presence of transposase in the 5′- and 3′-extremity of the cluster and its high nucleotide identity with gene clusters found in phylogenetically distant species such as *Enterococcus* and *Lactococcus* support that the ability to ferment galactose through the T6P pathway is a feature likely acquired by few STH strains. However, experimental verification is required to validate the ability of strains CIRM-BIA336 and JIM10116 to catabolize galactose throughout the T6P pathway.

### Production of short chain fatty acids

SCFAs with lactate are the main bacterial fermentation end-products from sugar metabolism. The major SCFAs are butyrate, propionate and acetate. In addition to their nutritive role, SCFAs modulate the inflammatory responses of epithelial and immune cells. SCFAs are formed in the gastro intestinal tract by bacterial fermentation of food fibers and resistant starch [88]. Numerous inflammatory gut diseases are linked to an imbalance of the intestinal microbiome, notably a decrease in the number of bacterial SCFA-producers [89]. Because SCFAs can be used as a fermentation source for other bacteria, supplementation with SCFA-producing bacteria is therefore a strategy to modulate the intestinal microbiome, increase endogenous SCFA production and restore dysbiosis [90, 91]. In agreement, it has been shown that a yogurt diet rich in acetate could improve the protective function of the intestinal epithelium [92]. Likewise STH strain ST4 increases the fecal content of acetate in correlation with maintenance of inflammatory homeostasis and preservation of intestinal permeability in a mucositis induced model [61].

Although this homolactic bacterium mainly produces L-lactate as the major fermentation end-product, it can also produce low levels of formate and acetate [17, 93]. From our analysis, all STH strains with complete genome sequence have the genetic determinants for the production from pyruvate of formate (Pfl, pyruvate-formate lyase; PflA, pyruvate-formate lyase activating enzyme) and acetate (Pta, phosphotransacetylase; AckA, acetate kinase) (Table S3). Three strains, however, could be defective in acetate formation due to the presence of a truncated form of Pfl or PflA. No gene coding for proteins involved in butyrate and propionate formation was found. Additional work is required to determine whether acetate from STH can have a beneficial effect on intestinal ecosystem as shown for formate that stimulates proto-cooperation in mixed culture.

### Production of bioactive peptides

The proteolytic system, which is associated with faster growth and milk acidification, has been widely studied for its essential role to sustain the growth of STH. Indeed, milk is poor in free amino acids and STH, like many other LAB, is multi-auxotrophic for amino acids [94–96]. This function contributes also to the formation of flavor and texture of the fermented milk products [17]. The proteolytic system that encompasses proteins involved in the breakdown and uptake of milk proteins and peptides, is notably composed of an extracellular proteinase, PrtS, responsible of milk protein degradation. In addition to its role in nitrogen supply, PrtS-induced degradation of bovine milk proteins liberates peptides with bioactive activity [97–99]. Most are angiotensin-I-converting enzyme inhibitors, but peptides with immunomodulatory, antioxidant and antibacterial activity can also be generated among others [99–101]. Although caseins are the main source of proteins and therefore of bioactive peptides in bovine milk, PrtS is also able to degrade β-lactoglobulin and α-lactalbumin, allowing to increase their digestibility and to hydrolyze β-lactoglobulin-derived allergenic peptides [102, 103]. Moreover, other protein sources such as goat milk and plant substrate have been successfully tested for bioactive peptides production by STH sole or in combination with other species [104–106]. Bioactive peptides can be produced during food fermentation by STH, but they can also be added as ingredients. Indeed, Paul and Somkuti, who studied the effect of yogurt starters on two bioactive peptides showed that they remained intact at acidic pH in the presence of STH suggesting that the addition of pre-formed bioactive peptides to yogurt near the end of fermentation is conceivable to produce a stable functional food [107].

**Fig. 4:**
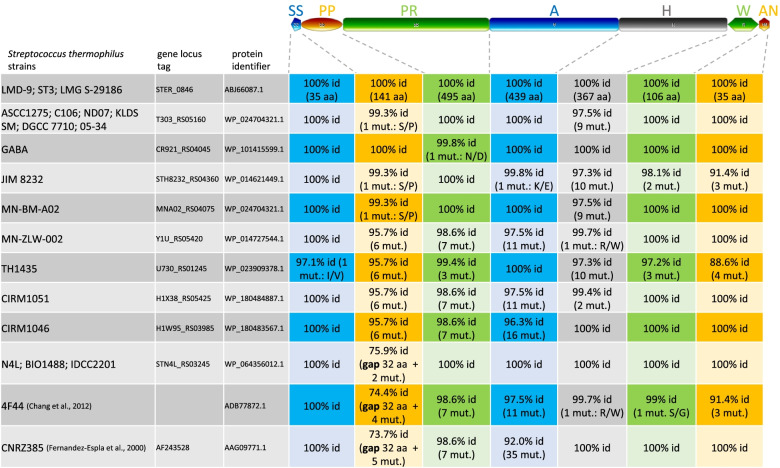
PrtS protease domain alignments and amino acid sequence identity percentages (reference: PrtS from STH LMD-9). The domains of PrtS are: SS, signal sequence; PP, propeptide; PR, catalytic domain; A, globular domain; H, helical domain; W, cell wall domain; AN, cell wall anchor. Domain length is indicated in number of amino acids (aa). The two biochemically characterized sequences of PrtS from strains CNRZ385 [113] and 4F44 [112] were added

*PrtS* was acquired by HGT from *Streptococcus suis* and the selection of fast acidifying strains has increased its prevalence in the STH population, notably among commercial starters [20, 108]. Therefore, according to the strains set screened, the number of strains containing the *prtS* gene, i.e., *prtS*^+^ strains, can vary from approximately 30 to 50% [20, 109–111]. Here, we found a complete *prtS* gene in 19 genomes, meaning that a maximum of 23% of the strains could be proteolytic (Table S3). Indeed, despite the presence of *prtS*, Galia et al. found that only 65% of *prtS*^+^ strains expressed measurable cell envelope-associated proteinase activity [110]. Among the 19 *prtS*^+^ strains, IDCC2201-, N4L- and BIO1488-derived PrtS exhibited a shorter sequence, due to the lack of an imperfect duplication of a 32-residues motif in the PP domain. This has already been reported for strain 4F44 [112] and CNRZ385. Nevertheless, although shorter, the protease expressed was still active in 4F44. In addition to the duplicated region, 56 variable positions over the 1618 amino acid sequence length of the 19 protein variants were detected corresponding to an average divergence over all sequence pairs of 0.96%. Fig. 4 shows the mapping of these variable positions according to the seven PrtS functional domains [113]. Variable positions were mainly located into the PP propeptide domain, the PR catalytic protease domain, the A globular domain involved in the regulation of the PrtS activity/specificity and the H helical domain. It is interesting to note that the pairwise distance between PrtS sequences from LMD-9 and 4F44 is around 1.6% and the two proteins are able to produce different bioactive peptides from caseins [99]. Whether the PrtS amino acid diversity, notably in the PR and A domains, is a contributing factor to the liberation of diverse bioactive peptides from milk proteins still needs further investigations.

### Production of GABA

GABA, a non-protein amino acid and a primary central nervous system neurotransmitter inhibitor, has been classified as a health-promoting metabolite with antihypertensive, antidiabetic, diuretic and tranquilizer effects [114]. Due to its function on human health, the development of functional foods containing GABA has attracted great interest [115]. Production of GABA has been reported in several major groups of LAB used in dairy fermentations, including STH, thus offering the opportunity to develop naturally fermented GABA-enriched products [65, 67, 114].

**Fig. 5:**
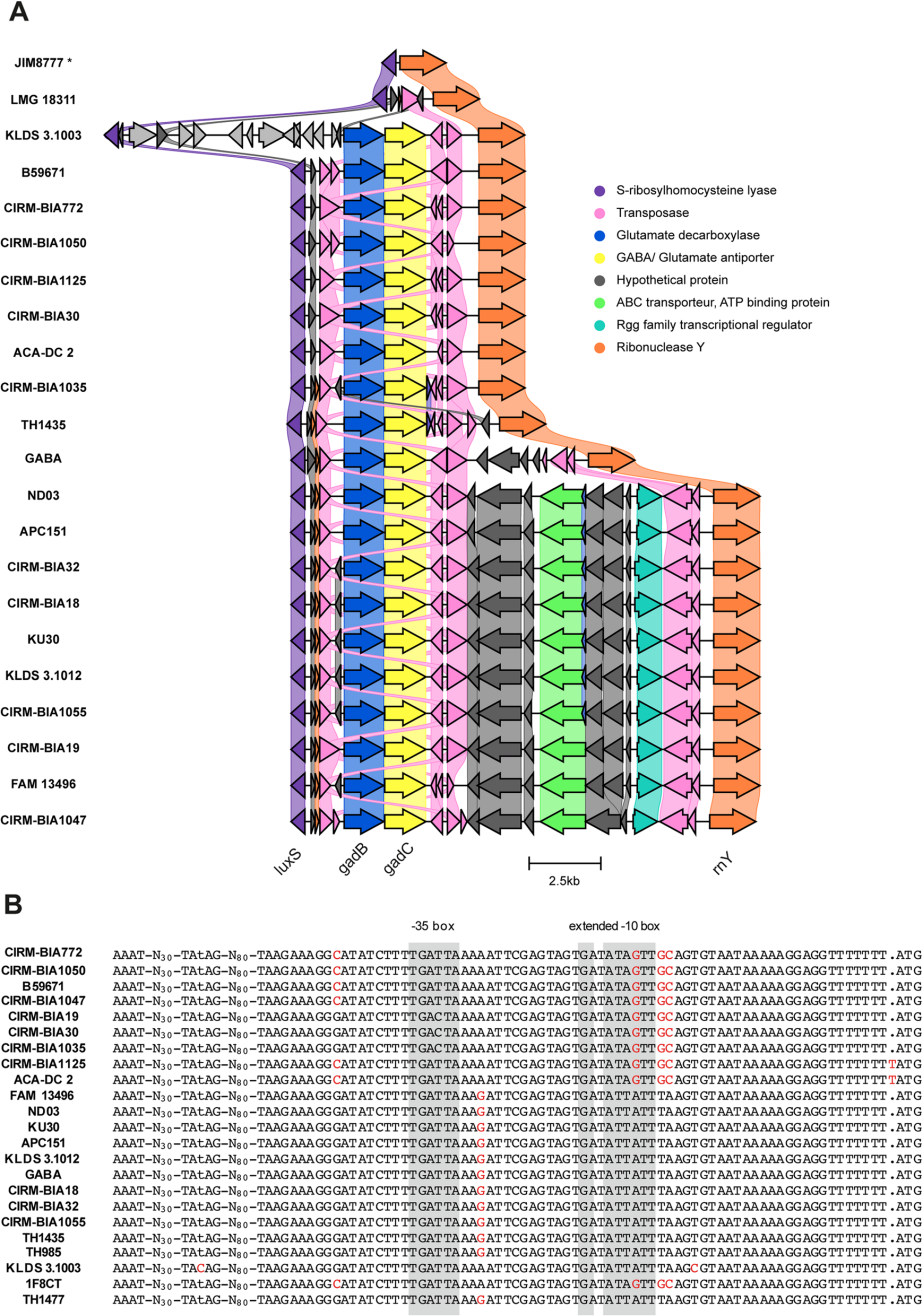
Genomic features of GABA biosynthetic genes in *S. thermophilus*. **A** Comparison of the genomic region harboring the *gadB* and *gadC* genes coding for GABA production in 21 *S. thermophilus* strains and *S. salivarius* strain JIM8777. The relative lengths of genes (pentagons) and intergenic regions are drawn to scale. *, strain not belonging to the species STH. The *gadBC* containing strains 1F8CT, TH985 and TH1477 were excluded from this comparative analysis due to the location of *gadBC* on too short contigs. **B** Nucleotide sequences alignment of the *gadB* promoter region of 23 *S. thermophilus* strains. The deduced − 35 and extended − 10 sequences are grey-boxed and variable nucleotides are in red

The glutamate decarboxylase enzyme (GadB, EC 4.1.1.15) synthesizes the irreversible conversion of glutamate into GABA, which is secreted throughout the GadC GABA/Glutamate antiporter. Twenty-nine percent of strains (23/79) contain both *gadB and gadC* that form a cluster of two contiguous genes (Fig. 5A, Table S3). This result is higher than previously reported (10%) and shown by a PCR-based screening of the presence of *gadB* on a set of 191 strains mainly isolated from Italian raw-milk cheeses [109]. The *gadBC* genes are surrounded by two copies of IS6 (IS1216) family transposase (98% nucleotide identity) and are part of a larger locus flanked upstream by *luxS* coding for AI-2 synthesis protein, and downstream by *rnY* coding for ribonuclease Y (Fig. 5A). The *gadBC*-containing locus, which is predicted as a region of genomic plasticity by the MicroScope database, appears to be variable in STH. It contains numerous IS transposases and specific genes in a strain-dependent fashion. A chromosomal region containing only genes *luxS* and *rnY* is present in *S. salivarius* and *S. vestibularis*, two species that share a common ancestor with STH [2]. This set of genomic data suggests that the *gadBC*-containing region tends to undergo multiple evolutionary events and that an ancestral strain may have acquired the *gadBC* genes by HGT. Accordingly, the polymorphism of *gadB* and *gadC* gene coding sequences is low with an average divergence over all sequence pairs of 0.3 and 0.8%, respectively (maximum divergence between sequence pairs of 1.0 and 1.5% for *gadB* and *gadC*, respectively). However, sequence comparison of the *gadB* upstream region between strains showed more nucleotide differences (average divergence over all sequence pairs of 1.6%, with a maximum divergence between sequence pairs of 3.6%). These differences are of particular interest, notably those located in the putative promoter region (Fig. 5B). Indeed, they could impact the *gadB* expression level and therefore the production of GABA. It has been reported that production of GABA can vary from 0.6 to 80 mg/kg of GABA according to strains [116]. Whether sequence variations in the *gadB* promoter region and *gadBC* coding sequences contribute to GABA production diversity between strains need further experimental investigation, but it could clearly be taken into account to select high GABA producers.

### Biosynthesis of folate and other B-group vitamins

Folate (vitamin B9) is involved, as a cofactor, in many essential functions of cell such as synthesis of nucleic acids and of amino acids, cellular growth and cell division. Folate deficiency is associated with a variety of human health disorders including osteoporosis, poor cognitive performance, Alzheimer’s disease, neural tube defects in newborns and colorectal and breast cancer [117]. Because humans cannot synthesize folate, this vitamin must be obtained from the diet. A wide variety of foods naturally contain folate, including seafoods, fruits, vegetables, eggs and milk. Interestingly, folate content of food products can be increased up to 20-fold after fermentation by folate producing bacteria such as STH [118–120]. Therefore, fermentation represents a tool to produce natural folate-enriched foods, particularly in fermented dairy products, which are considered as good sources of folate. Several studies have shown the ability of STH to produce folate extracellularly during growth in various media, including milk [121, 122]. However, the amount of folate accumulated was shown to depend on media, growth kinetics, culture conditions and individual strains [119, 120, 123].

In an attempt to better understand the causes of such strain-dependency in folate production, we first explored the diversity of folate synthesis at the genomic level. Folate is synthesized from the precursors GTP and *p*-aminobenzoate (PABA), which derive from the purine and phenylalanine metabolism, respectively (Fig. S3). Most STH strains harbor the genetic determinants for de novo folate synthesis (Table S3). However, as observed for most organisms, no gene specifying an alkaline phosphatase (EC 3.1.3.1) can be found on the genomes of STH and it has previously been proposed that this reaction is catalyzed by nonspecific phosphatases [124]. In *L. lactis*, the synthesis of dihydroneopteridine is performed throughout an alternative route catalyzed by a dihydroneopterin triphosphate diphosphatase (EC 3.6.1.67), a protein belonging to the large and functionally heterogeneous Nudix hydrolase family of enzymes [125]. Genomes of STH harbor several genes coding for putative Nudix enzymes but none of the corresponding genes are located within the *fol* cluster as shown in *L. lactis*. The involvement of a member of the Nudix enzyme family, a nonspecific phosphatase, or other enzymes in dihydroneopteridine synthesis remains to be elucidated. From these genomic data, the strain-dependent capacity to synthesize folate might not be related to the absence or inactivation of folate-specific genes but might result from metabolic bottlenecks in dihydroneopteridine synthesis. Variations in sequence composition of enzymes between strains could also lead to marked differences in folate production within the species STH. However, the polymorphism in folate biosynthetic enzymes is low with an average divergence over all sequence pairs from 0.3% for FolC2 to 1.2% for FolK (maximum divergence between sequence pairs of 6.8%). These divergences between sequences might however account for the folate production diversity among STH strains. Although it has been reported that the overexpression of *folK* in *L. lactis* increases the secretion of folate [123], the level of expression of the *folK* and *folP* genes in STH is not correlated with the amounts of folate secreted into the environment indicating that other mechanisms are involved [126]. These may include, for example, transcriptional control mechanisms, modulation by metabolites of metabolic flux and the amount of folate consumed or excreted by strains. Further studies are needed, notably genomic comparison between low and high folate excreters to understand the basis of STH strains to produce and secrete folate [57].

The capability of STH to synthesize the seven other B-group vitamins was also evaluated. All STH strains with complete genome sequence have incomplete de novo thiamin (vitamin B1), riboflavin (vitamin B2), pantothenate (B5), pyridoxine (vitamin B6) and biotin (vitamin B8) biosynthesis pathways (data not shown). This analysis confirms at a larger scale vitamin B2 and B5 auxotrophy of STH determined by single omission experiments [95, 127]. However, the presence of parts of the biosynthetic pathways suggests that STH can produce certain vitamins from extracellular intermediates provided by other bacteria in mixed cultures. For example, the presence of genes coding for pyridoxal kinase (EC 2.7.1.35), pyridoxamin-phosphate transaminase (EC 2.6.1.54) and pyridoxal 5′-phosphate synthase (EC 1.4.3.5) indicates that STH strains could produce pyridoxal 5-phosphate, the active form of vitamin B6, from pyridoxal, pyridoxamine and pyridoxamine 5′-phosphate (Table S3).

De novo vitamin B3 synthesis pathway was found complete only in strain M17PTZA496. However, two out of the three genes that are missing in most STH strains and coding for enzymes involved in synthesis of nicotinate from aspartate, NadA (EC 2.5.1.72) and NadB (EC 1.4.3.16) are truncated in strain M17PTZA496 and might thus result in nonfunctional enzymes. The *nadABC* locus of strain M17PTZA496 is present on a 20 kb contig (AZJT01000081) with 99% nucleotide identity to a chromosomal region in *S. macedonicus* ACA-DC 198 and *S. gallolyticus* ICDDRB-NRC-S1 suggesting this region was acquired by HGT in strain M17PTZA496. Although the ability of this strain to produce and excrete vitamin B3 remain uncertain, this finding points out that peculiar strains could produce other B-group vitamins than folate. Harnessing the natural STH biodiversity, therefore, could be source for innovation in the field of vitamin-enriched fermented products.

### Production of conjugated linoleic and linolenic acids

Conjugated linoleic acid (CLA) and conjugated linolenic acid (CLNA) are a group of isomers of linoleic acid and linolenic acid, respectively, that have been shown to exert numerous health benefits, including anti-carcinogenesis, anti-obesity, anti-diabetic and anti-inflammatory effects. Milk is the main source of CLA and CLNA in the human diet. Their concentration in cow milk, which is affected by lactation stage, rumen microbiota and dietary regime of cows can be enhanced by fermentation [128]. Several strains of LAB including STH have been shown to produce CLA and CLNA from their respective acids [129–132].

The conversion of linoleic and linolenic acid into their conjugated forms, CLA and CLNA, respectively, is carried out by linoleate isomerase (EC 5.2.1.5). To our knowledge, the genetic determinants involved in CLA/CLNA synthesis are unknown in STH. However, the genome of six strains (CIRM-BIA336, CIRM-BIA368, CIRM-BIA956, CIRM-BIA998, JIM10116 and M17PTZA496) encodes a protein annotated as oleate hydratase with homology (63–68% identity) to linoleate isomerase from *Limosilactobacillus reuteri* ATCC 55739, *Lactobacillus acidophilus* AS1.1854 and *L. lactis* KF147 [133] (Table S3). STH oleate hydratase proteins shared 98% identity with Sph protein from *Weissella cibaria* strains isolated from Kimchi and sourdough [134]. Although the CLA/CLNA-producing ability of these STH strains as well as the linoleate isomerase activity of the proteins identified remain to be demonstrated, they are promising candidates that can be explored to enhance CLA/CLNA contents in fermented dairy products.

### Production of bacteriocins

In their competition for nutrients, LAB produce metabolites including active antimicrobials such as lactic or acetic acid, hydrogen peroxide, and/or bacteriocins. Bacteriocins are ribosomally synthesized proteins with bactericidal activity. They are natural products with great potential in food biopreservation, against both pathogens and spoiling microorganisms. Bacteriocins usually exhibit antimicrobial activity towards strains closely related to the producer strain or towards other species sharing the same ecological niche [135]. STH strains can produce bacteriocins with inhibitory activity against both Gram-positive and Gram-negative bacteria including LAB as well as, among others, *Listeria monocytogenes*, *Enterococcus faecalis*, *Enterococcus faecium*, *Clostridium botulinum*, *Bacillus cereus*, *Staphylococcus carnosus, Salmonella typhimurium* and *Escherichia coli* [136–139].

The assessment of antimicrobial activity is carried out in vitro by methods related to the agar well diffusion test on Petri dishes from Tagg and McGiven [140] on selected strains. Softwares were also developed to predict in silico bacteriocin loci from genomes, such as antiSMASH [141–143] and BAGEL4 [144–146]. Although more than 20 strains of STH have been reported in the literature as bacteriocin producers, only five genomes of those strains were sequenced in August 2021: B59671 [147], LMD-9 [148, 149], KLDS 3.1003 [150], ST106 and ST109 [151].

**Fig. 6:**
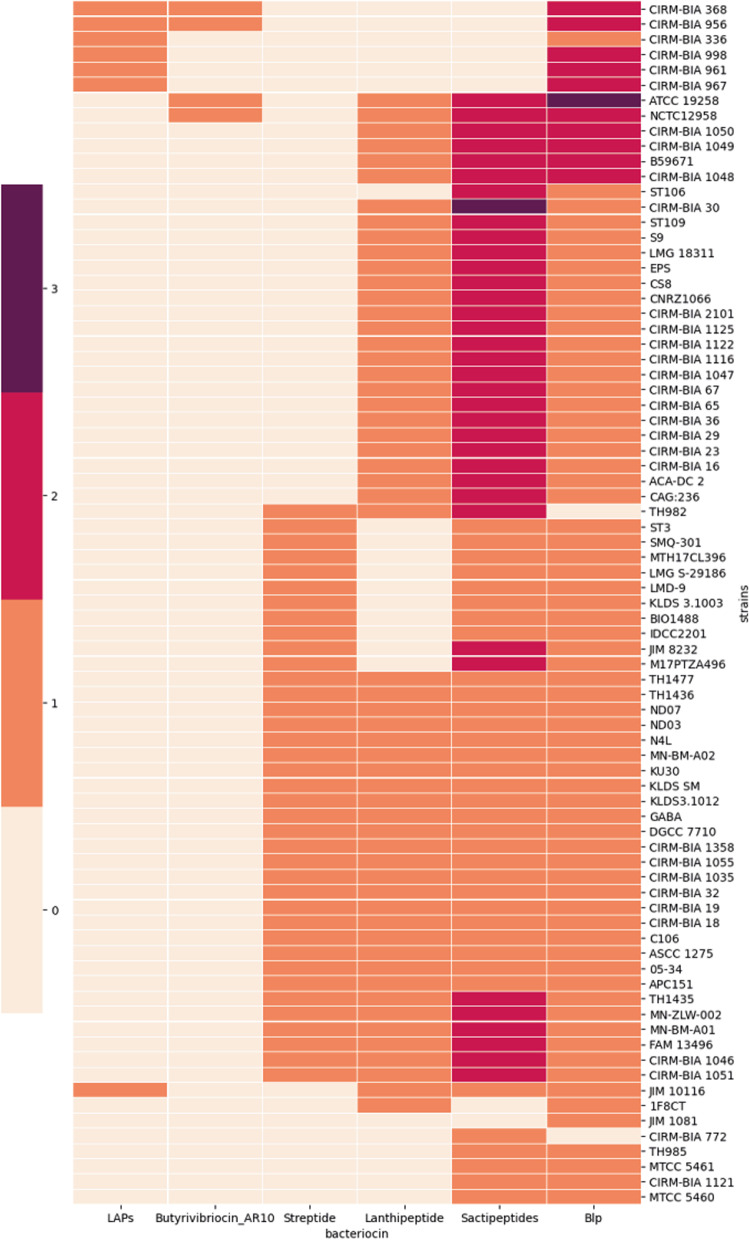
Heatmap of the presence or absence of bacteriocin clusters in 79 *S. thermophilus* strains (predicted by BAGEL4). The color gradient represents the number of predicted clusters per strain, from 0 to 4. LAPs: Linear azol (in)e-containing peptides

The results of bacteriocin prediction obtained in silico with BAGEL4 on the 79 genomes of STH investigated here are summarized in Fig. 6. From one to seven regions on the chromosome of each strain were annotated as potentially coding for bacteriocins including butyrivibriocin, lanthipeptides, streptide and Blp. In accordance to Hols et al. [17], the *blp* locus seems to be ubiquitous in STH strains, except for two strains: TH982 and CIMR-BIA772. These prediction results, nevertheless, are to be taken with precaution. Indeed, although a *blp* cluster was predicted in LMD-9, CNRZ1066 and LMG 18311 both by BAGEL4 and antiSMASH (data not shown), it has been shown that the *blp* cluster was only found to be functional in LMD-9 due to a truncated form of the BlpB protein on the two other strains, which is essential for the secretion of the induction factor BlpC [17, 149]. A similar truncated form of BlpB was found in the genomes of 18 strains, certainly leading to a non-functional *blp* cluster. Furthermore, the *blpF*, *blpE* and *blpD* genes are missing in most of the previous strains and eight strains also lack the two genes *blpQ* and *blpG*, leading to a very incomplete *blp* cluster (Table S4). Nevertheless, these strains could still exhibit an antimicrobial activity through a potential lanthipeptide, sactipeptide or streptide production. Tools for bacteriocin prediction in silico remain interesting to investigate potential antimicrobial strains, but up to date, in vitro experiments remain necessary to demonstrate the antimicrobial activity of a strain. Such standardized procedures would be interesting to compare the STH strains in terms of biopreservative potential. Nevertheless, STH species remains a promising source of antimicrobial peptides with a broad range of targets.

### Antioxidative activity

Antioxidative activity of LAB can confer a health benefit to the host by decreasing the risk of ROS (reactive oxygen species) accumulation [152] and therefore, can be considered as oxidative stress reducing supplements [153, 154]. STH exhibits antioxidative activity in vitro and in vivo*,* and prevents ROS-induced DNA damage in human cells [58, 155–157]. This function is mainly associated with its ability to produce ROS protective factors such as antioxidant compounds, mainly glutathione, and antioxidant enzymes that can strengthen the host antioxidant capability [156–158]. In addition to the major natural antioxidant peptide glutathione, other compounds from STH may exhibit antioxidant activity, including some already mentioned for their beneficial effects on human health such GABA, CLA, folate, EPS, and beta-lactoglobulin-released peptides [159, 160]. Glutathione synthetase catalyzes the formation of glutathione from glutamate, cysteine and glycine (Fig. S4). Antioxidant enzymes include superoxide dismutase and catalase as well as enzymes belonging to the glutathione and thioredoxin systems that play a role in hydroperoxide radical detoxification and redox cycle [161].

**Fig. 7:**
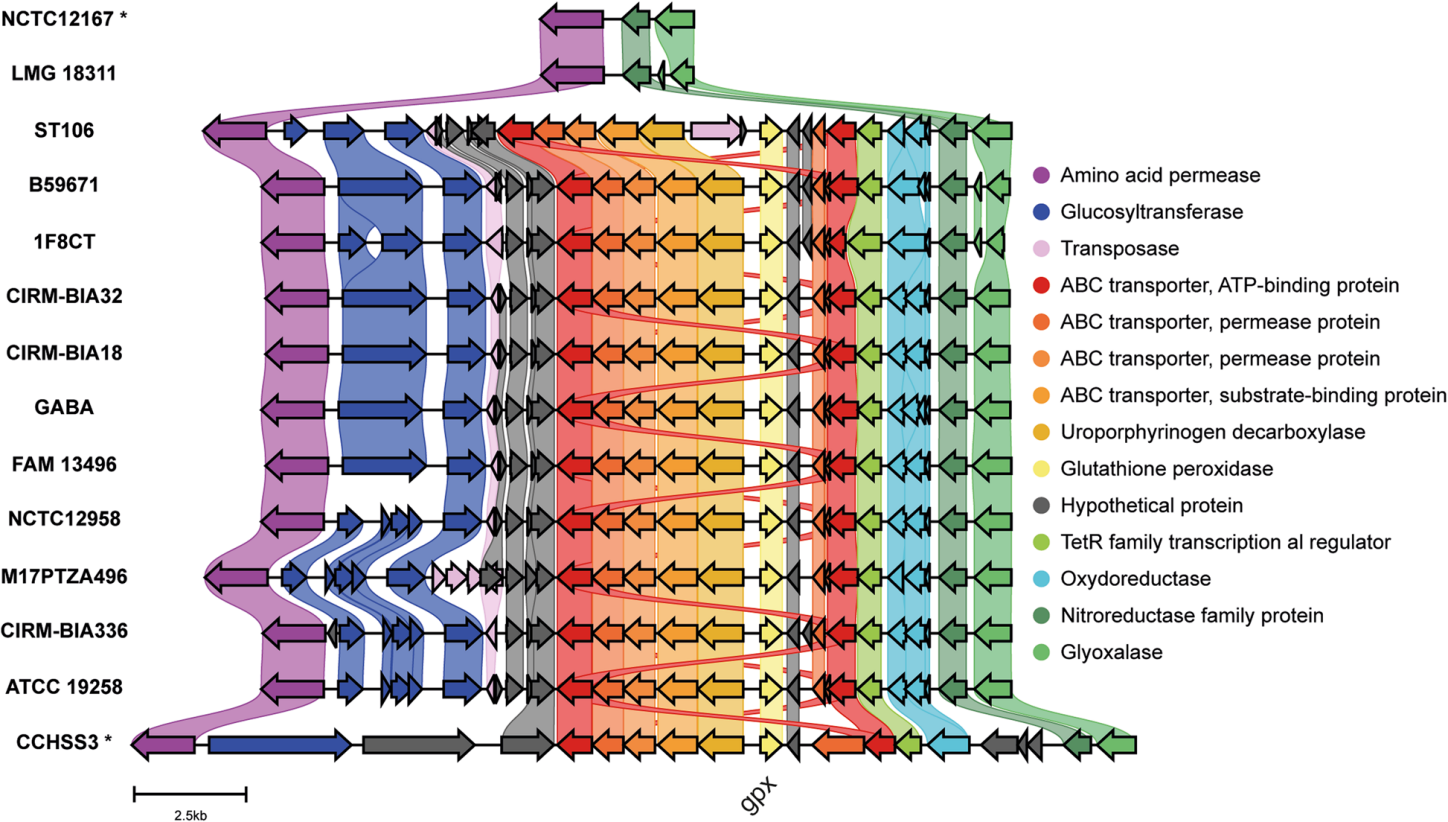
Comparison of the genomic region harboring the *gpx* gene in various *S. thermophilus* strains, *S. vestibularis* NCTC12167 and *S. salivarius* CCHSS3. *, strains not belonging to the species STH. The relative lengths of genes (pentagons) and intergenic regions are drawn to scale

Two genes coding for superoxide dismutase (SodA, EC 1.15.1.1) were found in all STH genomes, while no catalase was evidenced indicating that the detoxification of SodA-generated hydrogen peroxide is mainly achieved via the glutathione and thioredoxin systems. Glutathione synthase, glutathione reductase, glutathione peroxidase and glutaredoxin compose the glutathione system. The thioredoxin system includes thioredoxin reductase, thioredoxin-dependent peroxiredoxin and thioredoxin. Most STH genomes harbor a powerful arsenal of antioxidant enzymes (Table S3). One singularity detected, however, is the low occurrence of the *gpx* gene among strains (only 11 out of 79 genomes). The *gpx* gene codes for a glutathione peroxidase responsible of the reduction of hydrogen peroxide as glutathione is oxidized to its disulfide-oxidized form. Accordingly with this prediction, a glutathione peroxidase activity was reported in a very limited number of STH strains [158]. The *gpx* gene is included in a large gene cluster that also carries sulfur amino acid ABC transporter encoding genes (Fig. 7). The cluster is absent in all *gpx*-missing STH genomes, but present in *S. salivarius* genomes with high nucleotide identity (90%) suggesting its transfer between the two species or its loss in numerous STH strains. Overall, our analysis underlines the antioxidative potential of STH and its ability to provide ROS protective factors to host.

### Production of biogenic amines

Biogenic amines (BAs) are produced by many bacterial genera and species from the decarboxylation of amino acids [162]. They are found in varying concentrations in a wide range of foodstuffs such as fish and seafood, wine, meat and vegetables. In fermented dairy products, the most important biogenic amines are histamine, tyramine, cadaverine, putrescine and beta-phenylethylamine [163, 164]. The consumption of foods containing large amounts of BAs can have physiological and toxicological consequences including headaches, hypertension, edemas and various allergic syndromes such as asthma and urticaria [165, 166]. In addition to producing microorganisms, their formation is influenced by the raw material, processing technologies, and conservation conditions [162–164, 167]. One effective strategy to prevent harmful accumulation of BAs in dairy fermented products is to select LAB starters lacking BA biosynthetic activities [168] or with BAs degrading activities [169].

Like many LAB, STH has been described as potential BA producer, notably histamine and tyramine [163, 170–173]. A BLASTp-based search with histamine synthesis proteins from STH strain CHCC 6483 (embl accession no. FN686790.1) revealed that only a few STH strains (1F8CT, CIMR-BIA1121, CIRM-BIA1048, CIRM-BIA1049, TH1435 and KLDS 3.1003) contained genes coding for histamine production from histidine (Table S3). They are organized in a chromosomal gene cluster likely acquired through HGT that consists of three adjacent genes encoding the HcdA histidine decarboxylase proenzyme, the HdcP histidine/histamine antiporter, and HdcB, which is involved in HdcA maturation [174] (Fig. S5A). A satellite prophage harbors the *hdcABP* cluster in strain TH1435 [175]. Concerning tyramine production, no protein (BLASTp) and genomic region (tBLASTn) with homology to the TcdA tyrosine decarboxylase of STH strain 1TT45 [172] was detected in the 79 STH genome sequences. Likewise, no protein/genomic region with homology to ornithine decarboxylase (EC 4.1.1.17) (putrescine synthesis), lysine decarboxylase (EC 4.1.1.25) (tyramine synthesis), tryptophan decarboxylase (EC 4.1.1.105) (tryptamine synthesis) and phenylalanine decarboxylase (EC 4.1.1.53) (phenylethylamine synthesis) from various species was detected. These in silico predictions show that only a few STH strains contain BA-related genes. These findings are consistent with the previous results of a screening of 30 STH strains for the presence of histidine, lysine, and tyrosine decarboxylase genes by a PCR-based approach [25]. Ladero et al. reported that almost all the LAB that possess BA-related genes synthesize BAs at the phenotypic level [176]. This analysis shows that overall STH can be considered a species at low risk regarding biogenic amine production.

### Resistome and Virulome

The resistome of 79 STH genomes was investigated using ABRicate with multiple databases. Only two genes potentially conferring resistance to antibiotics were detected in nine strains. Four strains, B59671, CIRM-BIA1047, CIRM-BIA1050 and KLDS 3.1003, encode *mefE* which is 83% identical with 95% coverage at the nucleotide level with *mefE* of *Streptococcus pneumoniae*. The *mefE* gene codes for a proton efflux pump that confers resistance to macrolides including erythromycin [177, 178]. *S. pneumoniae mefE* gene is localized on a conjugative transposon-related element with *mel,* which is required for the maximal *mef*-mediated efflux of erythromycin [177]. No *mel* homologs were evidenced in STH genomes, suggesting that *mefE* is likely not able to confer high-level macrolide resistance in STH (Table S5). A *tet*(S) antibiotic resistance gene was detected in five other STH strains (CIRM-BIA368, CIRM-BIA998, CIRM-BIA967, CIRM-BIA961 and M17PTZA496) by four databases (NCBI AMRFinderPlus, Resfinder, ARG-ANNOT, CARD). Tet(S) confers resistance to tetracycline with a ribosomal protection protein [179]. The STH *tet*(S) gene is 100% identical with *tet*(S) from multiple species and is flanked by some mobile element proteins in STH genomes, which argue for an acquisition via HGT (Fig. S5B). Resistance to tetracycline of strain M17PTZA496 has been previously demonstrated suggesting that the four other *tet*(S)-containing strains are also tetracycline resistant [180]. The possibility of antibiotic resistance gene transmission from food bacteria to microbiota is a serious issue. However, no transfer of *tet*(S) from strain M17PTZA496 was detected due to its chromosomal location [180]. Finally, the protein sequences of all the strains under investigation were compared against the VFDB database to obtain data on potential virulence factors on the genome and no virulence factor were found. In all, the low occurrence of antibiotic resistance genes among STH strains contributes to the safety status of the species.

## Conclusions

The results of this comparative genomic study provide a sound basis for future studies on *S. thermophilus*. Notably our analysis provides targets for further phenotypic characterizations and experimental validations. While some beneficial traits are widely shared among isolates suggesting their central physiological and ecological role for the species (e.g., degradation of lactose, folate production), others including the tagatose-6-phosphate pathway involved in the catabolism of galactose, and the production of bioactive peptides and GABA are highly strain-specific. Most of strain-specific health-promoting features seems to have been acquired via horizontal gene transfer events. Likewise, sequence variations in the promoter region of certain genes from the core genome may be associated with the phenotypic diversity reported between strains for some beneficial traits. For instance, substitutions in the *galK* and *gadBC* promoter region could be associated with the ability of strains to catabolize galactose via the Leloir pathway and to produce high level of GABA, respectively. The low occurrence in the STH genomes of genes coding for biogenic amine production and antibiotic resistance is also a contributing factor to its safety status. Some isolates that distinguish themselves from others by combining health-promoting properties are promising candidates to develop functional and beneficial fermented food products. Knowledge associated with these features and their diversity between isolates may facilitate the selection and application of strains for specific biotechnological and health purpose. Moreover, with an open pan-genome, it is very likely that numerous STH strains not included in our analysis may have additional health-promoting properties that remain to be investigated. The decreasing cost of genome sequencing and the development of user-friendly and robust pipelines for genomic analyses are key factors to explore new health-promoting traits. The natural biodiversity of STH species is therefore an interesting source for innovation in the field of fermented products enriched in beneficial components for human health. In this context, its GRAS (Generally Recognized As Safe) and QPS (Qualified Presumption of Safety) status in the United States and the European Union, respectively, is of particular relevance. Moreover, until now, the development of fermented products by STH with beneficial effects on human health has mainly focused on dairy matrices. The ability of STH to ferment other matrices such as plant, fruit and vegetables based matrices could also be source of improved health effects, new health functionalities and of great innovation in this field. Finally, it is interesting to point out that STH is rarely used in single culture during fermentations, but mostly in mixtures with one or more other species, such as with *Lactobacillus delbrueckii subsp. bulgaricus* for yogurt production. The interactions between species during mixed fermentations that are not neutral can confer additional characteristics to fermented products compared to monocultures, which are at the origin of the diversity of their organoleptic and physicochemical properties. One can suppose that the interactions of STH with other species in mixed cultures can not only enhance its health-promoting properties but also elicit the production of additional health-promoting compounds notably via metabolic complementarity phenomena. The exploration and scientific knowledge of the interactions between species in mixed cultures will open new innovative avenues for developing new health promoting applications.

## Methods

### Genomes selection

Thirty one strains from the CIRM-BIA collection and two strains JIM1081 and JIM10116 (INRAE collection, Jouy-en-Josas, France) have been newly sequenced by Oxford Nanopore Technologies in our lab and by Illumina technology allowing an hybrid assembly [181]. All genomes were integrated in MaGe (MicroScope Genome Annotation) for annotation and comparisons (https://mage.genoscope.cns.fr/microscope/home/index.php) [182]. Furthermore, the 31 CIRM-BIA genomes were deposited in the European Nucleotide Archive (ENA, https://www.ebi.ac.uk/ena/browser/view/PRJEB36851) and they were made public in July 2020. In addition, genomes available in NCBI repository before June 2019 were selected and those with less than 150 contigs were kept, corresponding to 45 genomes: 27 complete, 8 chromosome, 3 scaffolds and 7 contigs. These genomes were also integrated in MaGe. One genome only available in MaGe, from strain CAG:236, was also added to this study. At all, 79 strains were selected (Table S1). To confirm that the selected genomes belong to strains from the same species, FastANI software was used to compute pairwise ANI (Average Nucleotide Identity) values among the 79 STH genomes [183]. The ANI values that ranged from 96.95 to 100% between a strain pair with a mean of 98.63 ± 0.63 confirmed that the 79 strains selected belong to the same species [184].

### Genomic features

MicroScope Pan-genome analysis tool was used to identify core, accessory, and unique protein families within 79 selected strains with the 80% amino acid identity and 80% alignment coverage option. Protein sequences were extracted and submitted to eggNOG-mapper to obtain COG classification of core and accessory genome [185, 186]. A presence / absence gene heatmap was created from accessory genome composition with R libraries pheatmap and dendextend [187]. Protein sequences of core proteome were gathered from Microscope and proteins duplicated in at least one strain were discarded. Sequences were concatenated strain by strain and aligned with MAFFT online version in auto mode [188]. Alignment was submitted to MEGA software to calculate a pairwise distance matrix and converted to a percentage difference matrix [189, 190]. Then the pairwise intersection heatmap was clustered with the linkage complete method on Euclidian distances and drawn with Intervene’s R library [191]. Annotations of pseudogenes from gene and product fields of MaGe annotation gff files were filtered to remove non informative words (protein, evidence(s), computational, gene, analysis, automated, derived, method, prediction, using, homology, derived, frameshifted, part, fragment, stop, type, from, function, internal, product, disrupted, pubmedid, experimental, organisms, other, incomplete, missing, partial, complete, genome, hypothetical, family, start, putative, subunit). Then word cloud was drawn from gene and product fields of MaGe annotation gff files with R libraries rtracklayer, tm, wordcloud and SnowballC [192, 193]. Fig. S6 showed the word cloud computed without pseudogene annotation filtering.

### Homolog search and analysis

The presence of some genes and proteins in the 79 STH genome sequences was investigated using tBLASTn and BLASTp searches implemented in the MicroScope platform as entry the following protein sequences: histamine decarboxylase from *S. thermophilus* CHCC6483 (FN686790.1); tyrosine decarboxylase from *S. thermophilus* 1TT45 (FR682467); ornithine decarboxylase from *Lactobacillus delbrueckii* subsp. *bulgaricus* (Q1G8S9), *Streptococcus pneumoniae* (A0A0B7M4H0), and *Œnococcus œni* (Q5ZH57); lysine decarboxylase from *Enterococcus faecalis* (Q838D6), *Enterococcus faecium* (A0A481NV25), *Lactobacillus brevis* (J7GQ11) and *Streptococcus pneumoniae* (A0A0H2ZRA0); tryptophan decarboxylase from *Ruminococcus gnavus* (A7B1V0), *Clostridium sporogenes* (J7SZ64) and *Flavobacterium* sp. 9R (A0A653PYX3); phenylalanine decarboxylase from *Enterococcus faecium* (Q1JTV5); linoleate isomerase from *Lactobacillus reuteri* ATCC 55739 (AX062045), *Lactobacillus acidophilus* AS1.1854 (DQ239438), *Lactobacillus plantarum* AS1.555 (DQ227322) and *L. lactis* KF147 (ADA64689.1); glutamate decarboxylase from *S. thermophilus* (Q0GE18); glutamate/gamma-aminobutyrate antiporter from *L. lactis* MG1363 (O30417); PrtS proteinase from *S. thermophilus* (D3KCP7, B9UZ65). MEGA X was used to align sequences and to calculate the divergence between sequence pairs and the average divergence over all sequence pairs using the maximum composite likelihood method [189, 190]. Sequences alignments of PrtS domains and promoter regions of *galK* and *gadB* were performed with ClustalW with default parameters. Gene clusters were aligned and drawn with clinker [194]. The proteinase PrtS domains were drawn with MyDomains [195].

### Resistome and virulome

ABRicate (https://github.com/tseemann/abricate) with NCBI AMRFinderPlus [196], Resfinder [197], ARG-ANNOT [198], CARD [199], EcOH [200] and VFDB [201] databases with default parameters (thresholds 75% identity with 0 coverage) was used to search antibiotic resistance and virulence genes in genome sequences. The softwares antiSMASH (accessible via MicroScope platform) [141] and BAGEL4 (URL: http://bagel4.molgenrug.nl/) were used to search bacteriocins and Ribosomally synthesized and Post translationally modified Peptides (RiPPs) clusters sequences in *S. thermophilus* genome sequences. Resulting heatmap was drawn with Python’s matplotlib library [202].

## Supplementary Information

**Additional file 1: Supplementary Figures**.

**Additional file 2: Supplementary Tables**.

## Data Availability

The datasets supporting the conclusions of this article are included within the article and its additional files.

## Abbreviations

ANI: Average Nucleotide Identity
BA: Biogenic Amine
CDS: Coding DNA Sequence
CLA: Conjugated Linoleic Acid
CLNA: Conjugated Linolenic Acid
COG: Clusters of Orthologous Groups
GABA: Gamma-aminobutyric acid
GRAS: Generally Recognized As Safe
GTP: Guanosine TriPhosphate
EPS: Exopolysaccharides
EFSA: European Food Safety Authority
HGT: Horizontal Gene Transfer
LAB: Lactic Acid Bacteria
PABA: *p*-aminobenzoate
QPS: Qualified Presumption of Safety
ROS: Reactive Oxygen Species
SCFA: Short Chain Fatty Acid
STH: *Streptococcus thermophilus*
T6P: Tagatose-6-Phosphate
