# Supplementary material for: The genomic basis of the Streptococcus thermophilus health-promoting properties
Source: BMC Genomics. 2022 Mar 16;23:210. doi: 10.1186/s12864-022-08459-y (PMC8925076; doi:10.1186/s12864-022-08459-y)
Supplement: Supplementary file 1 — Additional file 1: Supplementary Figures. [file 12864_2022_8459_MOESM1_ESM.docx]

**The genomic basis of the *Streptococcus thermophilus* health-promoting properties**

**Emeline Roux^1,2,3^, Aurélie Nicolas^1^, Florence Valence^1^, Grégoire Siekaniec^1,3^, Victoria Chuat^1^, Jacques Nicolas^3^, Yves Le Loir^1^, Eric Guédon^1*^**

^*^ Correspondence: eric.guedon@inrae.fr

^1^ INRAE, Institut Agro, STLO, Rennes, France

^2^ Université de Lorraine, CALBINOTOX, Nancy, France

^3^ Université de Rennes, INRIA, Campus de Beaulieu, Rennes, France

**
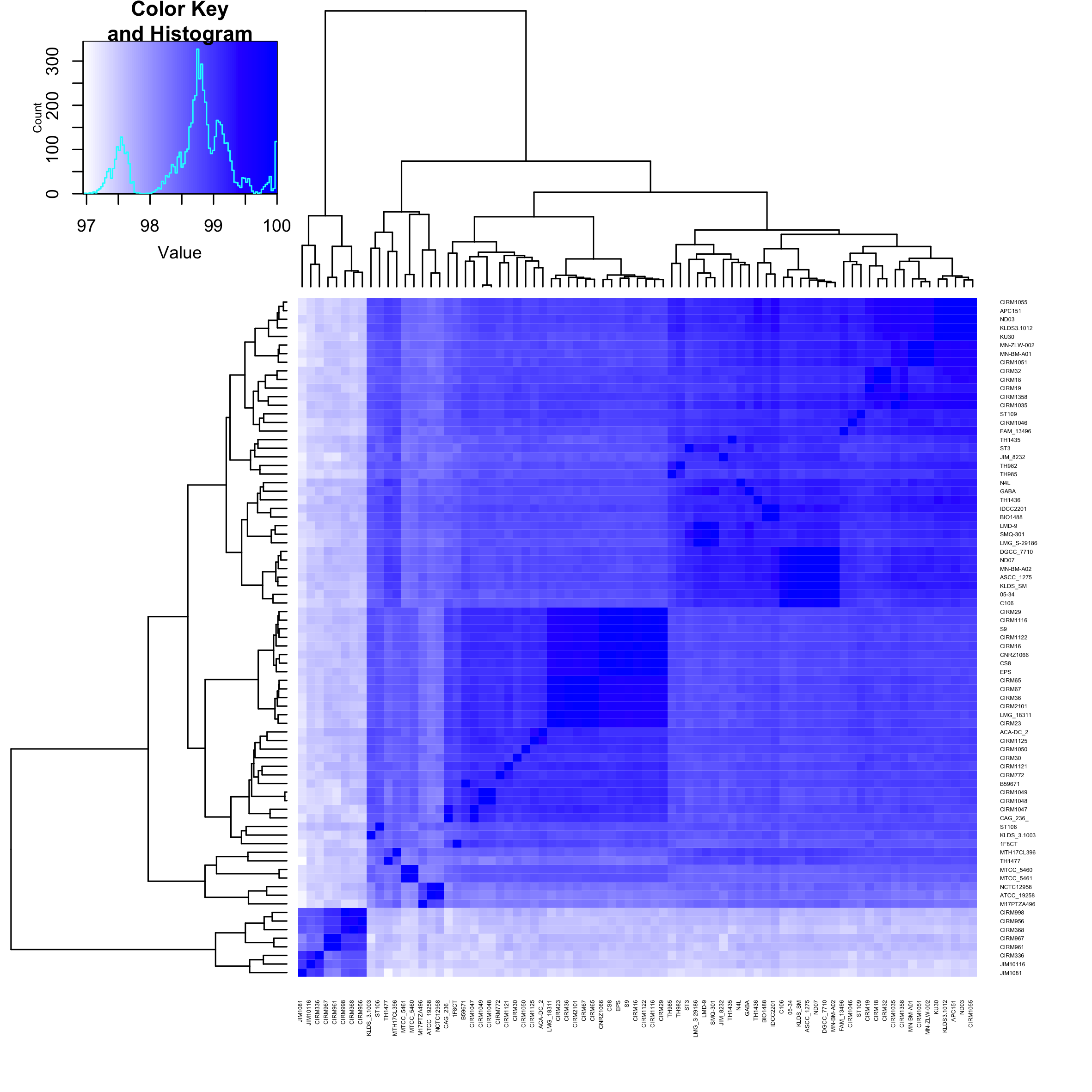
**

**Figure S1**. Whole genome Average Nucleotide Identity (ANI) pairwise heatmap of the 79 STH strains calculated with fastANI.

**
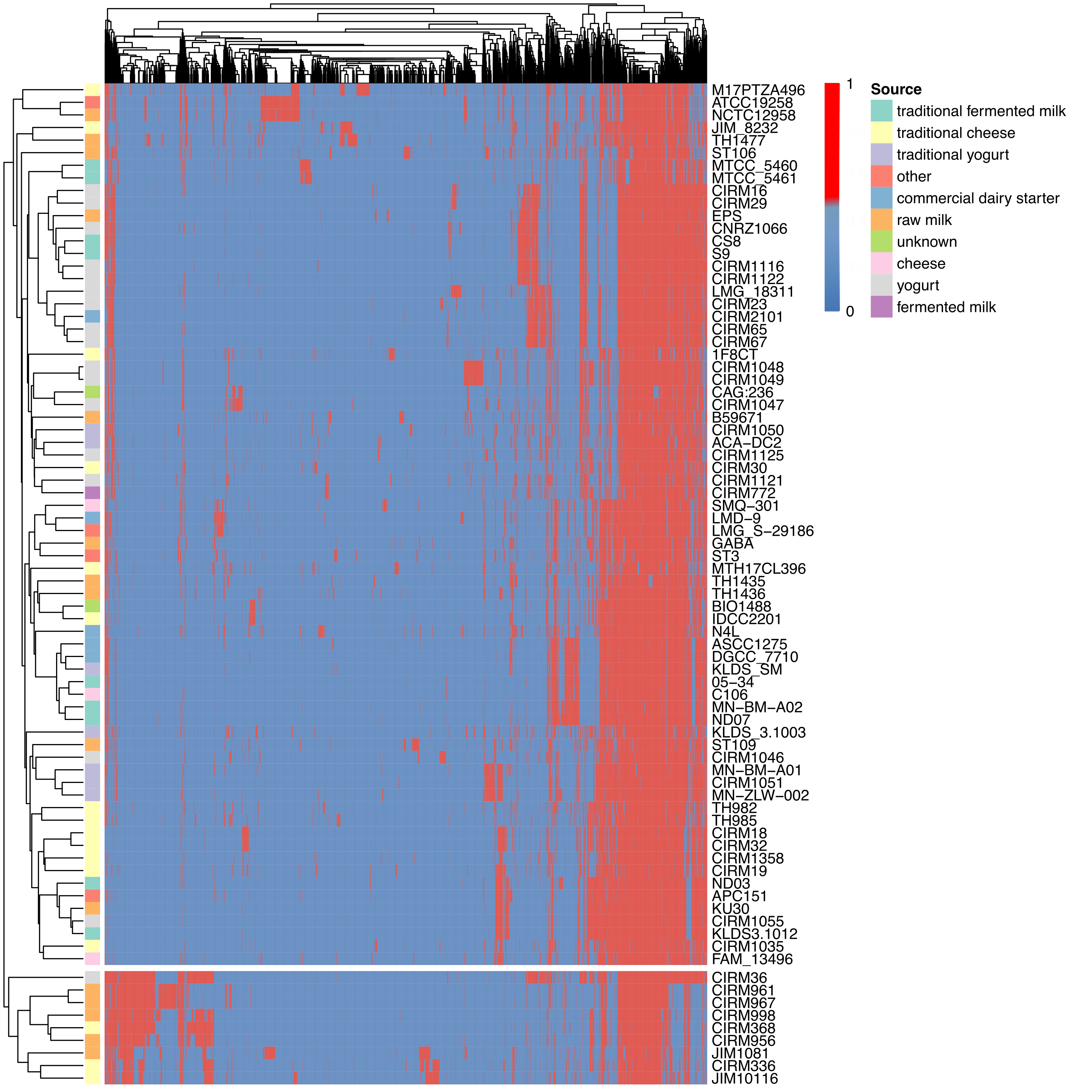
**

**Figure S2.** Heatmap of presence (red) or absence (blue) of accessory proteins in all strains. Strains are clustered in two groups. In the left column source ecosystem is indicated.

**Figure S3**. Folate biosynthesis pathway.

**Figure S4.** Redox metabolism in *S. thermophilus.*

**Figure S5**. Comparison of the genomic region harboring the **(A)** *hdcAPB* and **(B)** *tet*(S) loci in 6 and 5 *S. thermophilus* strains, respectively. The relative lengths of genes (pentagons) and intergenic regions are drawn to scale.

**
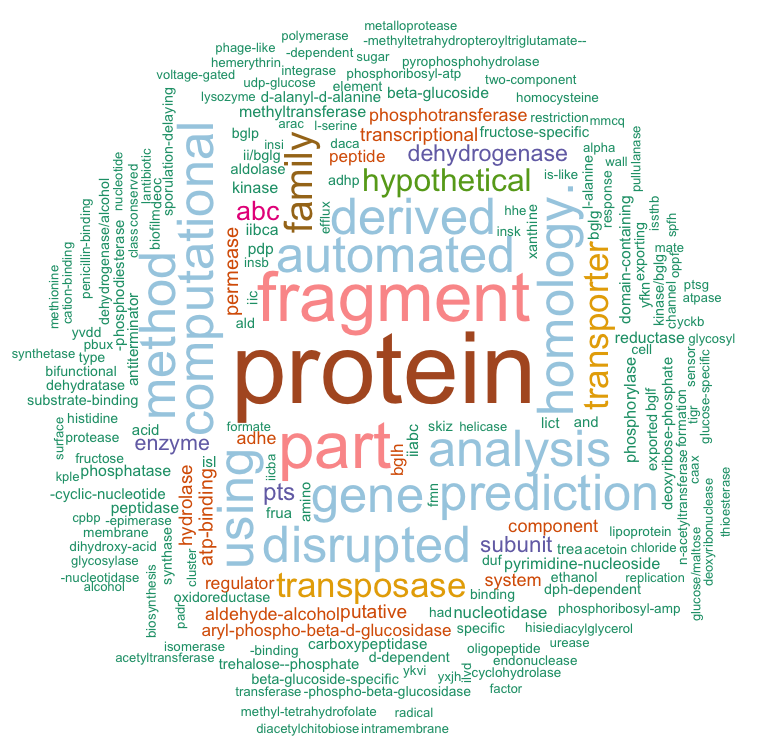
**

**Figure S6.** Raw word cloud of pseudogenes annotations from Microscope
